# Evaluating image-derived input functions for cerebral [^18^F]MC225 PET studies

**DOI:** 10.3389/fnume.2025.1597902

**Published:** 2025-06-05

**Authors:** Giordana Salvi de Souza, Pascalle Mossel, Joost F. Somsen, Laura Providência, Anna L. Bartels, Antoon T. M. Willemsen, Rudi A. J. O. Dierckx, Cristiane R. G. Furini, Adriaan A. Lammertsma, Charalampos Tsoumpas, Gert Luurtsema

**Affiliations:** ^1^ Department of Nuclear Medicine and Molecular Imaging, University of Groningen, University Medical Center Groningen, Groningen, Netherlands; ^2^ School of Medicine, PUCRS, Porto Alegre, Brazil; ^3^ Department of Radiology, Leiden University Medical Center, Leiden, Netherlands; ^4^ Department of Neurology, Ommelander Ziekenhuis Groep, Scheemda, Netherlands; ^5^ Laboratory of Cognition and Memory Neurobiology, Brain Institute, PUCRS, Porto Alegre, Brazil

**Keywords:** long axial field of view PET, pharmacokinetics, venous sampling, quantitative analysis, IDIF, kinetic analyses

## Abstract

Kinetic modelling of brain PET data is crucial for estimating quantitative biological parameters, traditionally requiring arterial sampling. This study evaluated whether arterial samples could be omitted to estimate the image-derived input function (IDIF) using a long axial field-of-view PET scanner. The use of internal carotid arteries (ICA) for IDIF estimation, along with venous samples for plasma-to-whole blood ratios and plasma parent fractions, was also assessed. Six healthy volunteers underwent [^18^F]MC225 scans with manual arterial sampling. IDIFs were derived from the aortic arch (IDIF_AA_) and calibrated using manual arterial samples (IDIF_AA_CAL_). ICA-derived IDIF was also calibrated (IDIF_CA_CAL_) and compared to IDIF_AA_CAL_. In a separate group of six volunteers, venous and arterial samples were collected to evaluate plasma-to-whole blood ratios, plasma parent fractions, and IDIF calibration (IDIF_CA_CAL_VEN_). Volume of distribution (V_T_) of different brain regions was estimated for all IDIFs techniques, corrected for plasma-to-whole blood ratio and plasma parent fraction (IDIF_AA,P_, IDIF_AA_CAL,P_, IDIF_ICA_CAL,P_ and IDIF_ICA_CAL_VEN_P_). Our findings revealed discrepancies between IDIF_AA_ and arterial samples, highlighting the importance of calibration. The differences between IDIF_AA,P_ and IDIF_AA_CAL,P_ were 9.2% for area under the curve and 4.0% for brain V_T_. IDIF_ICA_CAL,P_ showed strong agreement with IDIF_A_CAL,P_, with 1.2% V_T_ difference. Venous sampling showed consistent agreement with arterial sampling for plasma parameters but was unreliable for IDIF calibration, leading to 39% V_T_ differences. This study emphasises that arterial samples are still required for IDIF calibration and reliable V_T_ estimation for [^18^F]MC225 PET tracer. ICA-derived IDIF, when calibrated, provides reliable V_T_ estimates. Venous sampling is a potential alternative for estimating plasma parameters, but it is unsuitable for IDIF calibration.

**Trial Registry:**

NCT05618119 (clinicaltrials.gov/study/NCT05618119).

## 1 Introduction

Kinetic modelling translates dynamic positron emission tomography (PET) data into quantitative biological parameters. However, in the absence of a reference tissue, this usually requires an arterial input function (AIF)_,_ which most often involves continuous (or serial) arterial blood sampling (1). Manual arterial samples are used to measure the plasma-to-whole blood ratio and to quantify the fraction of the unmetabolised parent compound in plasma over time (2). Although this process is considered the gold standard, it is invasive, leading to associated patient discomfort. In addition, it requires specialised equipment and well-trained personnel (3).

For several applications, non-invasive quantification methods are used, e.g., for tracers with a reference tissue, when an image-derived input function (IDIF) can substitute the AIF, and when simultaneous estimation can be applied (1–5). Recent advances, such as long axial field-of-view (LAFOV) PET scanners, improve the feasibility of estimating whole blood time activity curves (BTAC) or IDIF from large vascular structures, such as the aorta or left ventricle (3, 4, 6–8). Earlier studies using standard short axial field-of-view (SAFOV) PET scanners successfully derived IDIFs from large vessels for tracers such as [^15^O]H_2_O and [^18^F]FDG (9–11).

Building on these foundations, recent studies with LAFOV PET imaging validated the feasibility of extracting IDIFs from different regions of the aorta for different tracers, including [^18^F]FDG, [^15^O]H_2_O, and [^18^F]DPA-714 (8, 12, 13). However, IDIFs must still be calibrated using manual arterial samples to scale the curves to absolute radioactivity concentrations measured with a gamma counter. For [^18^F]DPA-714, calibration using manual arterial samples had to be performed for a reliable IDIF estimation (8). Further studies expanded LAFOV PET applications for IDIFs extraction to other tracers, such as [^18^F]PSMA-1007 and [^18^F]florbetaben (14, 15).

Despite these advances, the limited availability of LAFOV PET scanners means that many brain PET studies must rely on SAFOV PET systems. In those cases, the internal carotid arteries (ICA) are often used as a blood pool region for IDIF estimation (16–18). However, as previously demonstrated, ICA should not be used without proper partial volume effect (PVE) correction for scanners with at least a 3-mm spatial resolution for [^18^F]FDG (7). Further correction techniques, such as recovery coefficient-based adjustments, model-based PVE correction, or calibration incorporating arterial and venous blood sampling, can help mitigate these inaccuracies (4, 16).

Regardless of the extraction method, accurate kinetic modelling requires the concentration of non-metabolized tracer within arterial plasma as the true input function. Converting a BTAC or IDIF into a (total) plasma time-activity curve involves using a plasma-to-whole blood ratio. Subsequently, the plasma time-activity curve must be adjusted for the plasma parent fraction to obtain the final IDIF. These adjustments typically require arterial blood samples (2, 4). In theory, venous or arterialised blood could yield concentrations comparable with arterial values, at least at later time points (19, 20). This would allow a less invasive and more patient-friendly quantification. Nevertheless, differences in blood composition and metabolite levels between venous and arterial samples can impact kinetic accuracy (21).

This study focused on [^18^F]MC225, a tracer used to evaluate P-glycoprotein (P-gp) function (22). P-gp is an efflux transporter that removes xenobiotics from brain endothelial cells into the bloodstream, limiting the permeability of many substrates and central nervous system drugs across the blood-brain barrier (22, 23). Furthermore, no reference tissue is available for [^18^F]MC225, which hinders non-invasive quantification.

Therefore, the study aimed to:

1. Assess the feasibility of directly estimating the IDIF from the aortic arch (AA) using LAFOV PET, and compare these estimates with manual whole blood samples (gold standard).
2. Validate the use of the internal carotid arteries (ICA) for IDIF estimation in LAFOV PET to assess its applicability to SAFOV PET.
3. Investigate whether venous samples can be used as an alternative to arterial samples to estimate the plasma-to-whole blood ratio and plasma parent fraction and calibrate the IDIF.

## 2 Methods

### 2.1 Participants

This study included two groups of participants. Six participants were scanned on a LAFOV PET/CT to validate IDIF estimation from the AA. The same individuals were used to validate the use of ICA to estimate the IDIF. Six other participants were acquired on a SAFOV PET/CT, with arterial and venous blood samples for plasma-to-whole blood ratio and plasma parent fraction estimation. A previous study showed that the performance of both scanners for brain imaging is comparable (24).

The study was approved by the Medical Ethics Review Committee of the University Medical Center Groningen (protocol ID 2022.555, NCT05618119). Written informed consent was obtained from all participants.

### 2.2 PET acquisition

[^18^F]MC225 was synthesised at the University Medical Center Groningen (EU-GMP production license: 108964 F), as previously described (23). Following a 60 s injection of 215 ± 72 MBq [^18^F]MC225 in the antecubital vein, 60 min of list mode data were acquired on either a Biograph Vision Quadra PET/computed tomography (CT) (Siemens Healthineers, Erlangen, Germany) or a Biograph Vision PET/CT (Siemens Healthineers, Erlangen, Germany).

List mode data were binned into 26 frames (1 × 10, 10 × 5, 1 × 10, 2 × 30, 3 × 60, 2 × 150, 4 × 300, 3 × 600 s) and reconstructed using an ordered-subset expectation maximisation algorithm (8 iterations, 5 subsets) with time-of-flight and point-spread function and a voxel size of 1.65 × 1.65 × 1.645 mm^3^. Data were corrected for attenuation, random coincidences, scattered radiation, dead time, and decay. Image reconstruction for Biograph Vision Quadra was performed using e7tools, a prototype research software package from Siemens Healthineers (Erlangen, Germany).

### 2.3 Magnetic resonance image (MRI) acquisition

A sagittal 3D T1w MPRAGE (repetition time of 2,300 ms, echo time of 2.31 ms, inversion time of 900 ms, flip angle of 8°, slice thickness of 0.9 mm, voxel size: 0.9 × 0.9 × 0.9 mm^3^) was acquired for all subjects. MRI scans were obtained as anatomical references for all PET scans. MRI scans were performed using a 3.0 T Magnetom Prisma (Siemens Healthineers, Erlangen, Germany) with a 64-channel head coil.

### 2.4 Arterial and venous blood sampling

For the subjects of the first cohort (LAFOV PET/CT), manual arterial whole blood samples were collected 5, 10, 20, 40, and 60 min after injection of [^18^F]MC225. These samples were used to measure plasma-to-whole blood ratio using a gamma counter (2,480 WIZARD 2, Waltham, PerkinElmer, USA), cross-calibrated against the PET scanner. The concentration, measured in Bq/g, was converted to kBq/ml using the whole blood density (1.06 kg/L) (25). As previously described, the plasma parent fraction was determined using thin-layer chromatography analysis with F-254 silica plates (Sigma-Aldrich, Germany) (23).

For the subjects scanned on the SAFOV PET/CT, manual samples were collected from the radial artery and the antecubital fossa vein opposite the injection site at 5, 10, 20, 40, and 60 min after tracer injection.

### 2.5 IDIF calibration

The IDIF was generated using PMOD PVIEW (*version* 4.0, PMOD Technologies Ltd., Zürich, Switzerland). Eight circular regions of interest (ROIs), each with a radius of 5 mm, were manually placed in consecutive slices centrally in the AA on an early summed PET image (50–80 s). These ROIs were combined into a single volume of interest (VOI), which was projected onto the dynamic image sequence to generate the IDIF, referred to as IDIF_AA_.

A hybrid approach, IDIF_AA_CAL_, was developed to adjust the IDIF_AA_ by incorporating manual arterial blood samples. A three-exponential fit (17) was applied to manual arterial samples, and concentration values were derived between 220 s and 535 s intervals, timeframes close to the peak. A calibration factor (CF) was calculated by averaging the ratio between the fitted concentration values (denoted as $\mathrm{Concentratio}n_{\mathrm{fit}})$ and the IDIF_AA_ values (denoted as $\mathrm{Concentratio}n_{\mathrm{AA}})\;$ over the interval from 220 s to 535 s, as represented by the equation:

$$\mathrm{CF}=\frac{1}{N}\overset{535s}{\underset{t=220s}{\sum }}\frac{\mathrm{Concentratio}n_{\mathrm{fit}}\;(t)}{\mathrm{Concentratio}n_{\mathrm{AA}}\;(t)}$$

where *N* denotes the total number of time points within the interval from 220 s to 535 s, $\mathrm{Concentratio}n_{\mathrm{fit}}$ is the concentration at time ***t***, derived from the three-exponential fit applied to the manual arterial blood samples and $\mathrm{Concentratio}n_{\mathrm{AA}}\;$ is the concentration at time ***t***, extracted from the IDIF_AA_.

**Figure 1:**
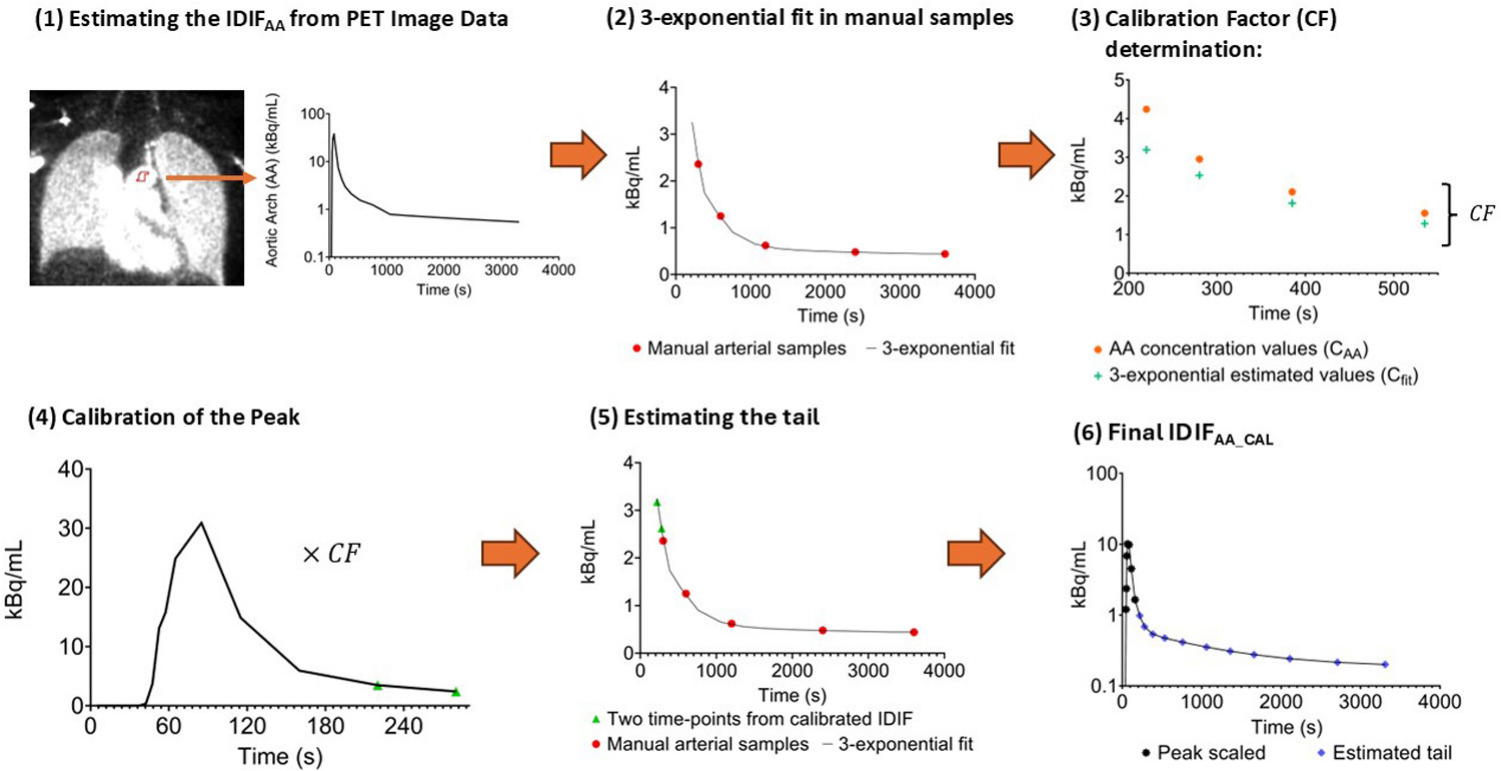
Overview of the methodology for estimating IDIF_AA_CAL_. **(1)** Generation of the initial IDIF (IDIF_AA_) by placing circular ROIs in the aortic arch on an early PET image. **(2)** A 3-exponential fit is applied to manual arterial samples, and concentration values are derived between 220 s and 535 s intervals. **(3)** The fitted concentration and IDIF_AA_ values are compared between 220 and 535 s to calculate the calibration factor (CF). **(4)** The CF is applied to scale the early part of IDIF_AA_ (0–280 s). **(5)** The tail is estimated using a 3-exponential fit to the manual arterial samples, incorporating the concentration values at 220 s and 280 s. **(6)** The final IDIF_AA_CAL_ is created by combining the scaled peak with the re-estimated tail.

The CF was then applied to correct the peak portion of the raw IDIF (0–280 s). For the tail portion of the curve, covering the interval from 220 s to 3,600 s, the previously calibrated PET-derived concentration values (at 220 s and 280 s) were combined with additional manual arterial sample data. A 3-exponential fit was applied to this combined dataset to estimate the mid-frame concentration values. Finally, the adjusted peak and the fitted tail were merged to form the calibrated IDIF_AA_. The methodology to generate the IDIF_AA_CAL_ is summarised in Figure 1.

### 2.6 Plasma corrections

IDIF_AA_ and IDIF_AA_CAL_ were multiplied by the plasma-to-whole blood ratio curve, which was obtained by fitting an exponential function to manual arterial sample data, to apply this correction. Moreover, the plasma parent fraction was fitted to a Hill function, as previously described (22). The resulting IDIF_AA_P_ and IDIF_AA_CAL_P_ curves include both plasma-to-whole blood ratio and plasma parent fraction corrections, with “P” indicating the application of both corrections.

### 2.7 IDIF for internal carotid artery

Sixteen circular ROIs, each 5 mm in radius, were manually placed in consecutive slices in the ICA on an early summed PET image (50–80 s). No partial volume correction was applied in the PET images. Four maximum pixel values from each ROI were determined and combined into a VOI to extract the concentration values for IDIF_ICA_ (7, 26). Next, IDIF_ICA_CAL_P_ was obtained using the same method as IDIF_AA_CAL_P_.

### 2.8 Venous sampling validation

Similar to the procedure for IDIF_ICA_CAL_P,_ IDIF_ICA_CAL_VEN_ was obtained using whole blood concentrations from the venous samples for IDIF calibration. In addition, plasma-to-whole blood ratio and plasma parent fraction were also estimated using venous samples using the same method described above. IDIF_ICA_CAL_VEN_P_ was derived, corrected by plasma-to-whole blood ratio and plasma parent fraction derived from venous samples.

### 2.9 Brain PET quantification

Motion correction was applied to all subjects using rigid transformation with the first 15 frames as a reference. PET images were co-registered to the individual anatomical T1-weighted MRI and spatially normalised to the Montreal Neurological Institute space using PNEURO PMOD (*version* 4.0, PMOD Technologies Ltd., Zürich, Switzerland). Brain regions of interest were defined based on Hammer's maximum probability atlas (27), including occipital, orbitofrontal, parietal, temporal cortices, cerebellum, whole brain white (WM), and grey (GM) matter.

### 2.10 Pharmacokinetic analysis

Pharmacokinetic analysis was performed using PKIN PMOD (*version* 4.0, PMOD Technologies Ltd., Zürich, Switzerland). Weighting factors based on time frame duration and decay were applied to the tissue TACs. Blood delay was estimated by fitting the first 10 min of whole brain GM data to a one-tissue compartment model. Regional tissue TACs were fitted to a reversible two-tissue compartment model, with fractional blood volume (V_b_) included as a fitting parameter and delay fixed to the estimate obtained for GM, yielding the volume of distribution (V_T_) as an outcome measure (22, 28).

### 2.11 Statistical analysis

Descriptive statistics are presented as mean ± standard deviation (SD). Statistical analyses were conducted using GraphPad Prism software (Boston, USA). The area under the curve (AUC) was used to compare IDIF estimations from different methods. The correlation was assessed using Pearson correlation analysis, and correlation (*r*), slope, intercept, and intraclass correlation coefficient (ICC) values were reported. Bias and agreement between methods were assessed using Bland-Altman plots, and the relationship between V_T_ values was quantified using linear regression analysis. For all methods that estimated V_T_, the percentage of difference was calculated.

## 3 Results

To facilitate understanding of the various input functions and calibration methods used in this study, we summarise the relevant acronyms and their definitions in Table 1. This table outlines the different IDIFs derived from the AA and ICA, the corresponding calibration approaches, manual arterial and venous blood sampling, and adjustments for plasma-to-whole blood ratio and plasma parent fraction.

**Table 1:** Summary of acronyms and descriptions of IDIF and calibration methods.

Label	Description
IDIF_AA_	Image-derived input function from the aortic arch (AA)
IDIF_AA_CAL_	IDIF_AA_ calibrated using manual arterial blood samples
IDIF_AA_P_	IDIF_AA_ corrected for plasma-to-whole blood ratio and plasma parent fraction
IDIF_AA_CAL_P_	IDIF_AA_CAL_ corrected for plasma-to-whole blood ratio and plasma parent fraction
IDIF_ICA_CAL_	IDIF from the internal carotid artery (ICA) calibrated with manual arterial blood samples
IDIF_ICA_CAL_P_	IDIF_ICA_CAL_ corrected for plasma-to-whole blood ratio and plasma parent fraction
IDIF_ICA_CAL_VEN_	IDIF from the ICA calibrated using venous whole blood samples
IDIF_ICA_CAL_VEN_P_	IDIF_ICA_CAL_VEN_ corrected for plasma-to-whole blood ratio and plasma parent fraction estimated using venous samples

### 3.1 Comparison of IDIF_AA_P_ and IDIF_AA_CAL_P_

**Figure 2:**
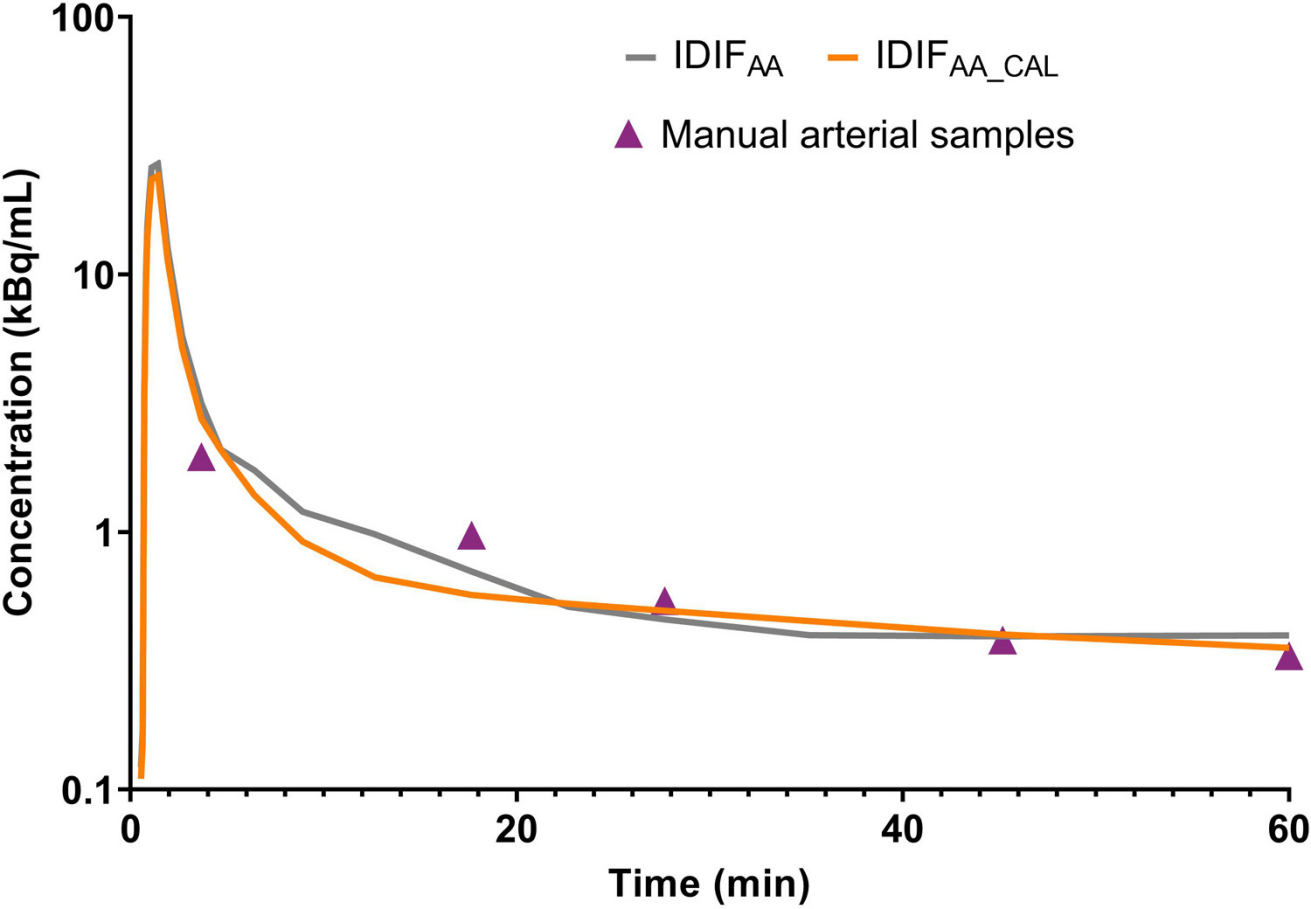
Comparison of IDIF_AA_, IDIF_AA_CAL_ and manual arterial samples for one subject (S05).

**Figure 3:**
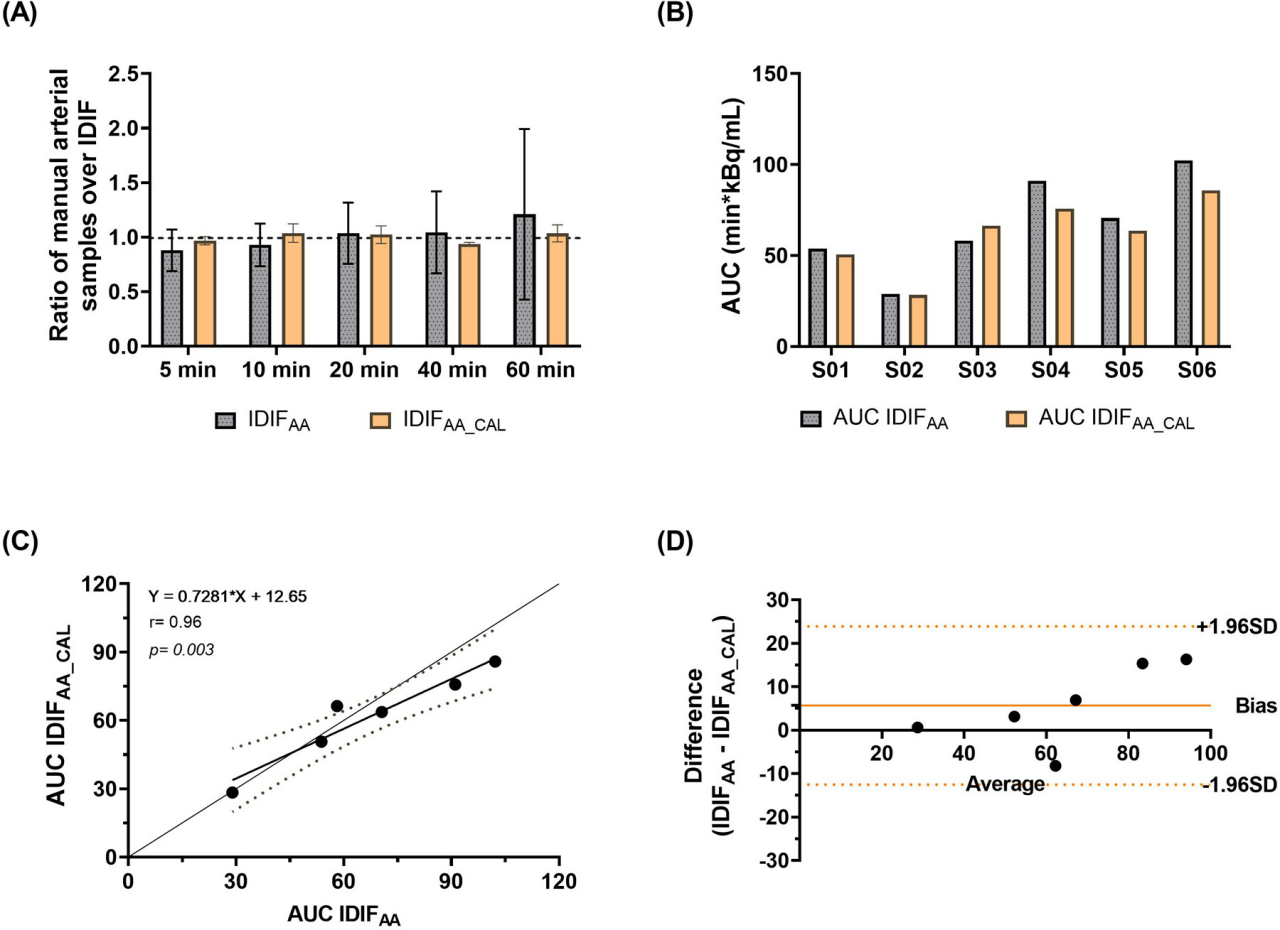
Evaluation of IDIF derivation methods. **(A)** The ratio of manual arterial samples to IDIF_AA_ and IDIF_AA_CAL_ values. **(B)** Comparison of AUCs for the two IDIF methods. **(C)** Correlation between IDIF_AA_ and IDIF_AA_CAL_. **(D)** Bland-Altman plot assessing the degree of agreement between the approaches.

Figure 2 illustrates an example of IDIF_AA_ and IDIF_AA_CAL_ from the same subject, comparing it with the manual arterial samples. To assess the difference between the IDIFs values and manual arterial samples, the ratio of manual arterial samples to IDIF_AA_ and IDIF_AA_CAL_ was calculated. These results are shown in Figure 3A. The AUCs of both IDIFs were estimated and compared (Figure 3B), with a mean AUC difference of 9.2%. Correlation (Figure 3C) and Bland-Altman plot (Figure 3D) were calculated. There were issues with blood sampling, plasma-to-whole blood ratio and metabolites estimation. In the Supplementary Material, all the reasons are explained. Individual V_T_ values across brain regions using IDIF_AA_P_ and IDIF_AA_CAL_P_ are provided in Supplementary Material Figure S1. Table 2 summarises the quantitative analysis of V_T_ differences, including bias, slope, Y-intercept, and ICC for the regions evaluated. Despite the minor differences in AUC and V_T_ values, the correlation between IDIF_AA_P_ and IDIF_AA_CAL_P_ ranged from 0.74 to 0.85, and the ICC ranged from 0.60 to 0.78, indicating that calibration improves the accuracy of the derived IDIF.

**Table 2:** Comparison of V_T_ values across subjects obtained using IDIF_AA_P_ and IDIF_AA_CAL_P_.

Brain regions	Mean % of difference V_T_	Bias	Slope	Y-intercept	*r*	ICC
Orbitofrontal Cortex	3.7%	0.20 **±** 0.94	0.51	2.6	0.83	0.74
Temporal Cortex	5.5%	0.30 ± 1.1	0.48	2.6	0.82	0.72
Parietal Cortex	3.1%	0.16 ± 0.85	0.46	2.8	0.78	0.68
Occipital Cortex	1.9%	0.11 ± 0.87	0.48	2.7	0.79	0.70
Cerebellum	2.8%	0.16 ± 0.93	0.54	2.5	0.85	0.78
Whole Brain GM	3.9%	0.21 ± 0.94	0.49	2.6	0.81	0.72
Whole Brain WM	7.4%	0.29 ± 0.82	0.38	2.2	0.74	0.60
Average ± SD	4.0 ± 1.7%	-	0.48 ± 0.05	2.57 ± 0.17	0.80 ± 0.03	0.71 ± 0.05

### 3.2 Validation of ICA

The comparison between IDIF_AA,_ IDIF_AA_CAL_, and IDIF_ICA_CAL_ is presented in Supplementary Material Figure S2, where ICA was validated as a surrogate for IDIF_AA_CAL_ across six participants. Individual V_T_ values across brain regions using IDIF_AA_CAL_P_ and IDIF_ICA_CAL_P_ are provided in Supplementary Material Figure S3. Table 3 compares V_T_ values across various brain regions derived using IDIF_ICA_CAL_P_ and IDIF_AA_CAL_P_.

**Table 3:** Comparison of mean V_T_ estimates for IDIF_ICA_CAL_P_ *vs.* IDIF_AA_CAL_P_ across different brain regions.

Brain regions	Mean % of difference V_T_	Bias	Slope	Y-intercept	*r*	ICC
Orbitofrontal Cortex	−1.5%	−0.08 ± 0.21	1.02	−0.03	0.98	0.99
Temporal Cortex	−0.9%	−0.05 ± 0.20	1.02	−0.04	0.98	0.99
Parietal Cortex	−1.6%	−0.08 ± 0.20	1.03	−0.10	0.97	0.98
Occipital Cortex	−1.6%	−0.09 ± 0.21	1.00	0.07	0.97	0.98
Cerebellum	−1.2%	−0.07 ± 0.22	1.05	−0.20	0.98	0.99
Whole Brain GM	−1.3%	−0.07 ± 0.21	1.03	−0.08	0.98	0.99
Whole Brain WM	−0.7%	−0.03 ± 0.14	1.07	−0.25	0.98	0.99
Average ± SD	−1.2 ± 0.3%	-	1.03 ± 0.02	−0.07 ± 0.10	0.98 ± 0.00	0.99 ± 0.00

**Figure 4:**
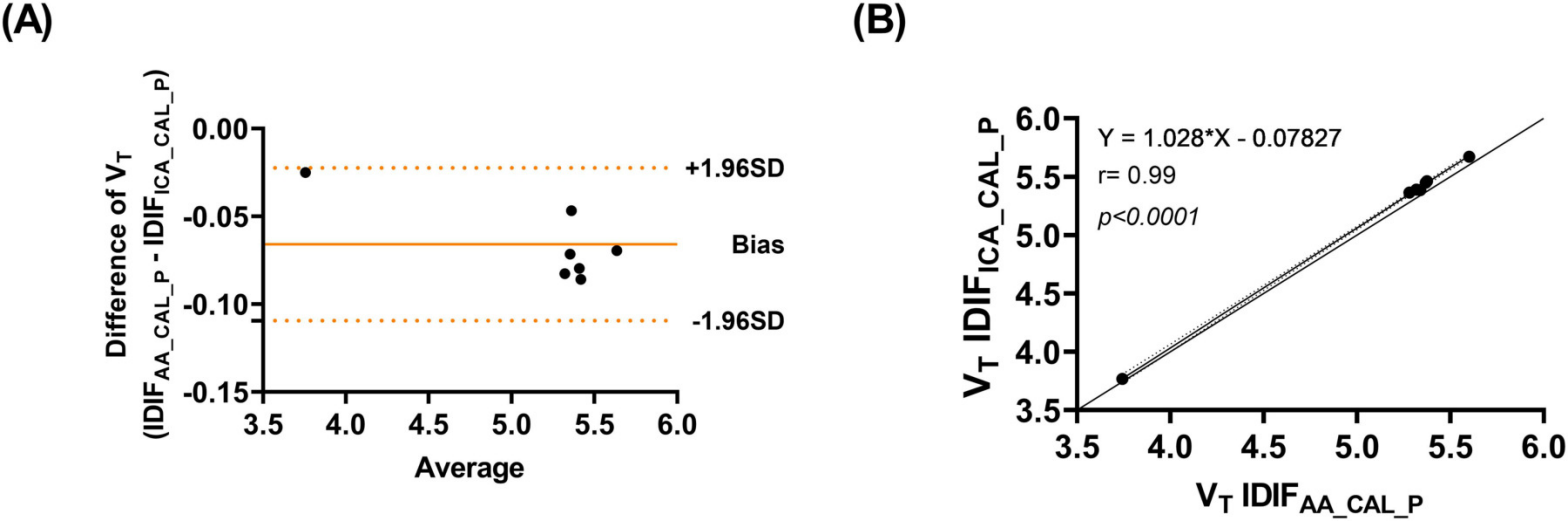
**(A)** Bland-Altman plot comparing V_T_ values estimated using IDIF_ICA_CAL_P_ and IDIF_AA_CAL_P_ **(B)** correlation of V_T_ values estimated using IDIF_ICA_CAL_P_ and IDIF_AA_CAL_P._

The Bland-Altman analysis (Figure 4A) demonstrates agreement between the two approaches, with minimal bias and low variability. Figure 4B presents the correlation analysis (*r* = 0.99*, p* < 0.001), with a mean V_T_ difference of 1.2% across brain regions and an ICC of 0.99, underscoring the high consistency between IDIF_ICA_CAL_P_ and IDIF_AA_CAL_P_. In addition, Supplementary Material Figure S4 presents an example of whole brain grey matter TACs from subject SO5, fitted using the three IDIFs evaluated: IDIF_AA_P,_ IDIF_AA_CAL_P_, and IDIF_ICA_CAL_P_.

### 3.3 Validation of venous samples

**Figure 5:**
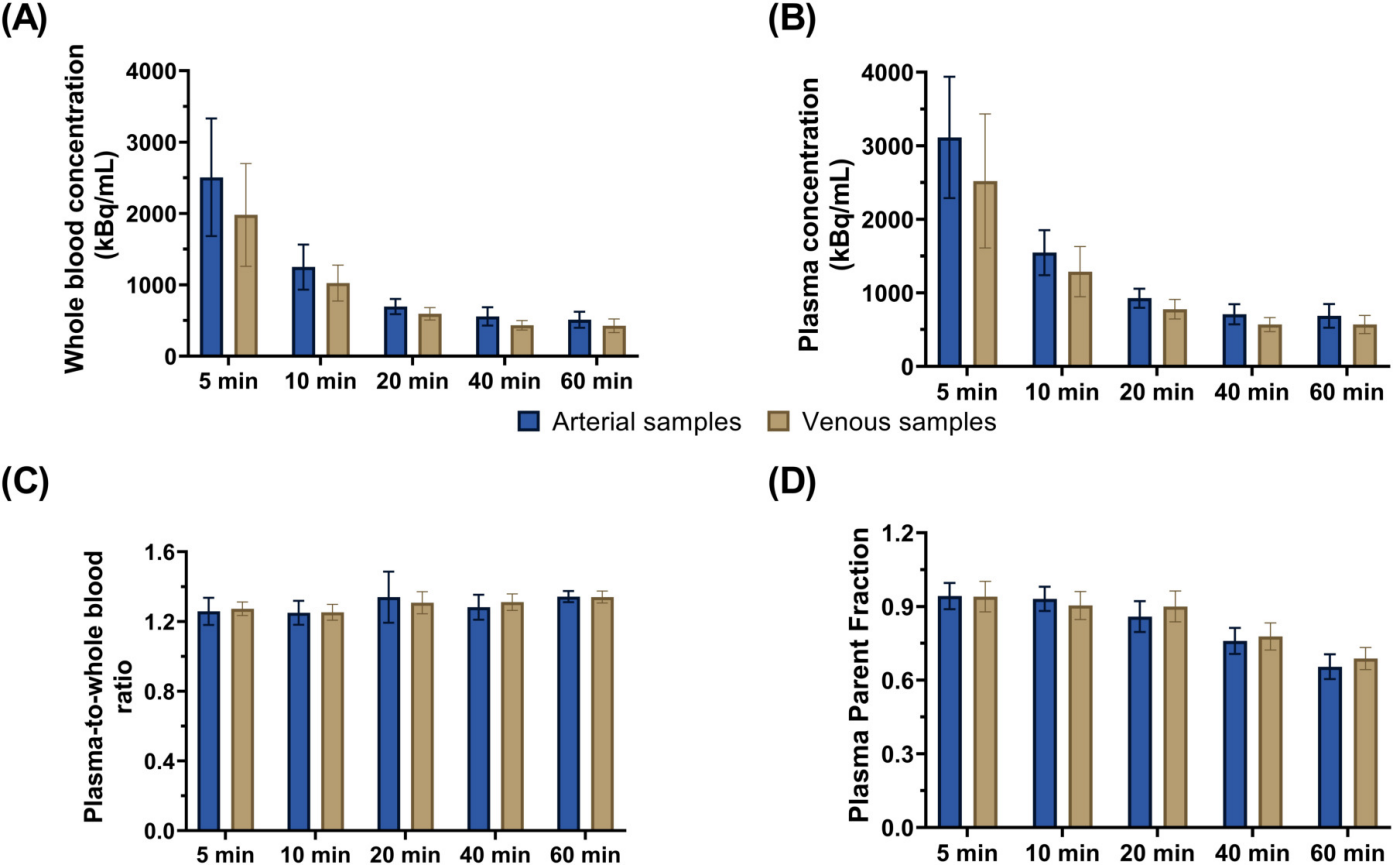
Concentration values from arterial and venous samples for **(A)** whole blood and **(B)** plasma. Comparison of arterial and venous manual samples for **(C)** plasma-to-whole blood ratio and **(D)** plasma parent fraction. Error bars represent standard deviation.

**Figure 6:**
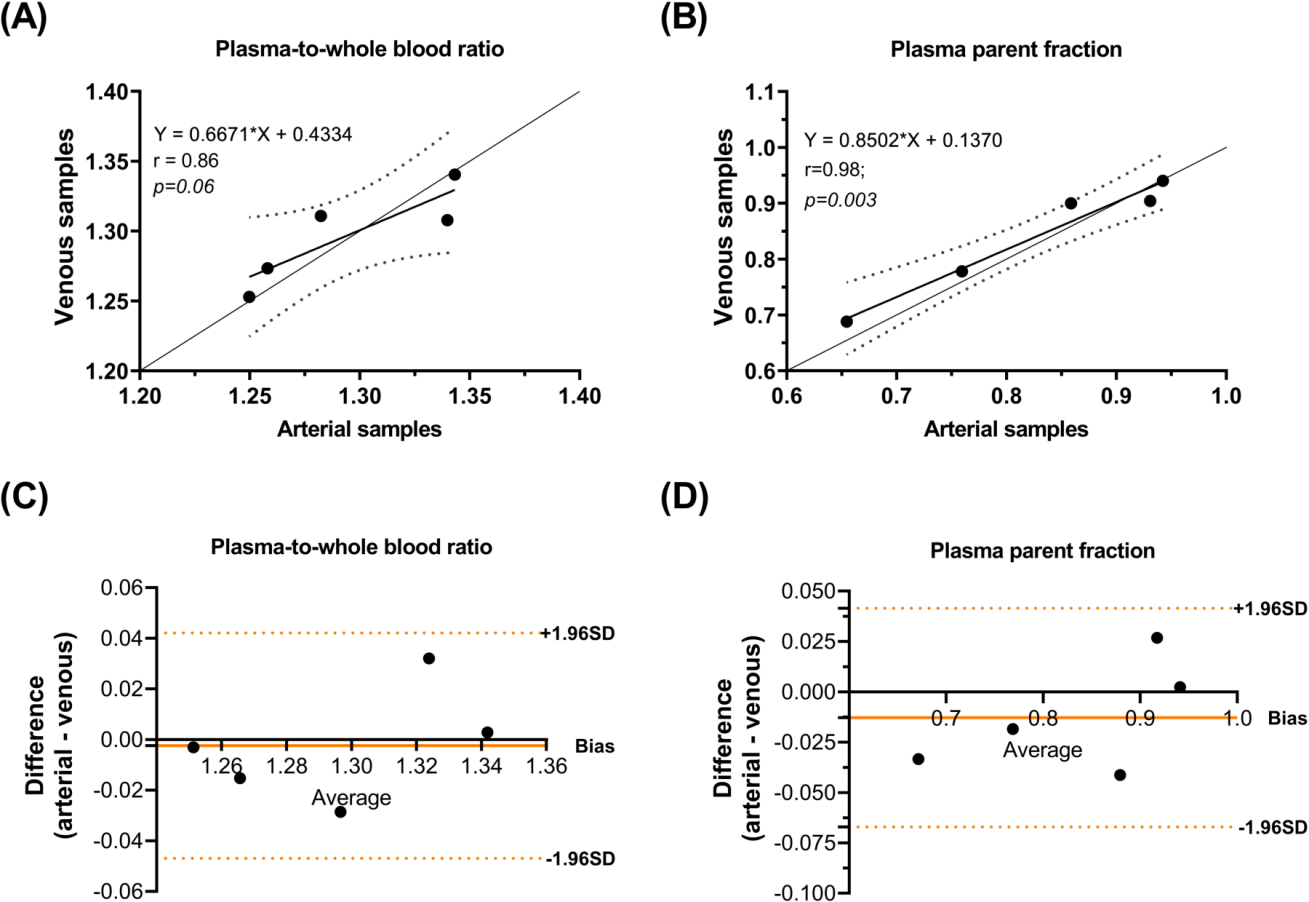
Correlation of **(A)** plasma-to-whole blood ratio and **(B)** plasma parent fraction. Bland-Altman plot differences between arterial and venous samples for **(C)** plasma-to-whole blood ratio and **(D)** plasma parent fraction.

**Figure 7:**
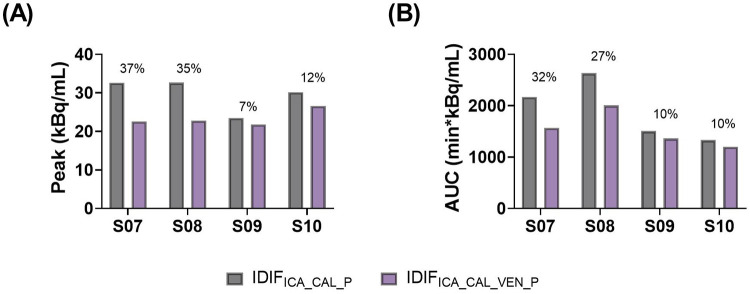
Comparison of **(A)** peak and **(B)** AUC values from IDIF_ICA_CAL_P_ and IDIF_ICA_CAL_VEN_P._ The percentage numbers represent the percentage difference between both methods.

Comparisons of whole blood, plasma concentrations, plasma-to-whole blood ratio, and plasma parent fraction values obtained with arterial and venous samples are shown in Figure 5. Higher variability in the whole blood and plasma concentrations was found during the first 5 min, both in arterial and venous samples. Figure 6 presents the correlation and Bland-Altman plots comparing arterial and venous samples for plasma-to-whole blood ratio and plasma parent fraction. Each dot in the Bland-Altman plot represents a time point. To assess the accuracy of the IDIF, the peak and AUC values were compared between IDIF_ICA_CAL_P_ and IDIF_ICA_CAL_VEN_P,_ as illustrated in Figure 7_._ From six subjects, two individuals, IDIF_ICA_CAL_VEN_P,_ could not be estimated, and V_T_ was not estimated: the first subject did not have plasma parent fraction estimated with venous samples, and the second subject had missing venous samples at the 5 and 60 min. For the third subject (S10), the 5 min sample was missing, but the analysis was conducted using the remaining venous samples. The V_T_ values for various brain regions were compared using the IDIF_ICA_CAL_P_ and IDIF_ICA_CAL_VEN_P_ to evaluate the reliability of venous sampling for calibration_._ The comparison presented in Table 4, highlights the percentage difference in V_T_ values between the two IDIFs across different brain regions. Calibration of IDIF derived from the ICA using venous samples (IDIF_ICA_CAL_VEN_P_) did not yield reliable V_T_ estimates, with discrepancies as large as 39% compared to IDIF_ICA_CAL_P_.

**Table 4:** Percentage difference in V_T_ between IDIF_ICA_CAL_P_ and IDIF_ICA_CAL_VEN_P_ across different brain regions.

V_T_ (ml·cm^−3^)				
Brain regions	S07	S08	S09	S10
Orbitofrontal cortex	−38.7%	−28.6%	−15.6%	−12.8%
Temporal cortex	−38.5%	−28.7%	−14.0%	−12.2%
Parietal cortex	−38.9%	−29.1%	−14.0%	−11.6%
Occipital cortex	−39.8%	−29.6%	−14.3%	−11.8%
Cerebellum	−38.9%	−29.0%	−14.2%	−12.4%
Whole brain GM	−38.8%	−28.9%	−14.2%	−12.3%
Whole brain WM	−36.9%	−27.3%	−13.4%	−9.4%
Average ± SD	−38.6 ± 0.8%	−28.7 ± 0.7%	−14.2 ± 0.6%	−11.8 ± 1.0%

Supporting figures are provided in the Supplementary Material. Supplementary Material Figure S5 compares whole blood TACs between IDIF_ICA_CAL_ and IDIF_ICA_CAL_VEN_. The resulting IDIFs derived from these curves are shown in Supplementary Material Figure S6. The impact of these differences on regional V_T_ values is illustrated in Supplementary Material Figure S7, and a representative example of grey matter TAC from subject SO7, fitted using both IDIFs, is shown in Supplementary Material Figure S8.

## 4 Discussion

This study evaluated whether IDIF derived from the AA using LAFOV PET requires additional calibration with manual arterial samples for [^18^F]MC225 PET tracer. Moreover, the use of ICA was assessed as a surrogate for AA due to its accessibility in brain PET imaging, particularly in SAFOV PET/CT scanners. To address the limitations of performing arterial sampling, venous sampling was explored as a less invasive alternative to reduce patient discomfort and simplify the blood collection process.

Comparison between IDIF_AA_ and manual arterial samples revealed differences in tracer concentration (Figure 3A). IDIF_AA_ underestimated concentrations at early time points (e.g., 5 min) and overestimated them at late time points (e.g., 60 min). This overestimation is likely attributable to PVE - spill-in from adjacent tissues, such as the myocardium and lungs, which showed high tracer concentrations (29–31). In addition to PVE, scatter correction and reconstruction algorithm settings may complicate accurate aortic concentration estimations (4, 8, 29). These differences underscore the challenges in achieving accurate corrections, calibrations, and image reconstruction settings.

As expected, IDIF_AA_CAL_ achieved ratios close to 1. The mean AUC difference between IDIF_AA_ and IDIF_AA_CAL_ was 9.2%, and the mean V_T_ difference was 4.0%. Despite the low AUC and V_T_ differences, the correlation ranged from 0.74 to 0.85, and the ICC ranged from 0.60 to 0.78; these metrics do not fully validate the use of IDIF_AA_ alone. Calibration improved the reliability of the IDIF_AA_ by aligning the measured tracer concentrations from manual arterial samples with those from the image-derived. This correction helped reduce discrepancies in tracer concentration estimates, especially in the early and late phases of the IDIF (Figure 2).

In addition, the use of ICA was explored as an alternative to using AA for IDIF estimation. The results showed that IDIF_ICA_CAL_ yielded highly consistent results when calibrated similarly to IDIF_AA_CAL_. Bland-Altman analysis confirmed minimal bias and low variability between the two methods, with a mean V_T_ difference of 1.2% across brain regions. The ICA-based approach achieved a high mean correlation (0.98) and ICC (0.99), indicating its reliability as a surrogate for the AA-derived input function.

Furthermore, the feasibility of venous sampling was investigated as an alternative to arterial sampling, similar to previous studies investigating venous sampling for other tracers (21, 32–34). For [^18^F]MC225, good agreement in plasma-to-whole blood ratio and plasma parent fraction was found between venous and arterial samples, as shown in the Bland-Altman plot (Figure 6). However, calibrating IDIF_ICA_ using venous samples (IDIF_ICA_CAL_VEN_P_) did not provide reliable estimates of V_T_, with differences up to 39% compared with IDIF_ICA_CAL_P_. These discrepancies may stem from the differences in whole blood concentrations between venous and arterial samples at 5 min time-point (Figure 5A), which likely influenced IDIF calibration. The arterio-venous equilibrium can explain the difference in this time-point (16, 33). While venous sampling shows promise for less invasive protocols, arterial samples remain essential for accurate V_T_ estimation. Enhancing venous sampling techniques or incorporating correction methods might improve its utility in less-invasive quantification.

These findings have significant implications for PET imaging research and clinical practice. Validation of AA-derived IDIF emphasises the need for manual arterial sampling to ensure reliable kinetic analysis for [^18^F]MC225. Moreover, the validation of ICA for IDIF estimation offers a practical alternative for brain imaging studies, particularly in SAFOV PET scanners. This approach could be extended to other tracers, enabling (limited) discrete arterial sampling rather than continuous sampling protocols. Although limited to V_T_ estimation, venous sampling presents a potential pathway toward reduced invasiveness for estimating plasma-to-whole blood ratio and plasma parent fraction. These findings contribute to developing patient-friendly scan protocols that may enhance compliance and streamline clinical trial procedures. To eliminate the need for arterial samples for calibrating the IDIF, further research must understand how venous sampling can be optimised, how to calibrate the IDIF, and how venous sampling can be optimised and potential errors can be addressed, particularly at early time points (e.g., the first sample). If omitting the first sample reduced the discrepancies observed, this could lead to more accurate calibration without arterial sampling, although this would need validation.

A key limitation of this study is the relatively small sample size (*n* = 6 per group), which restricts the statistical power of our analysis and limits the generalizability of our findings. However, given the nature of the study, increasing the sample size is unlikely to change the overall conclusion that calibration remains essential for reliable input function estimation. Furthermore, occasional missing data points due to issues with manual blood sampling may have introduced variability in the calibration and estimation of input functions. Future research should focus on developing and validating less invasive alternatives to arterial sampling, such as population-based corrections for the plasma-to-whole blood ratio and parent fraction. Their implementation in research settings requires rigorous validation in patient cohorts to ensure equivalence in quantitative outcomes. Furthermore, a better understanding of why calibration remains essential for certain tracers may inform the design of more accurate non-invasive methods. This could include advancements in image reconstruction algorithms and improved spillover correction strategies. Other strategies, such as single-point blood sampling or Simultaneous Estimation of the Input Function (SIME), also warrant further exploration. SIME offers a fully non-invasive framework for input function estimation by leveraging data across multiple brain regions without requiring direct invasive measurements (2, 5, 35, 36). It may represent a viable alternative to traditional arterial sampling in the context of PET quantification.

In conclusion, this study demonstrated that IDIF derived from LAFOV PET images requires calibration with manual arterial samples for accurate V_T_ estimation. While ICA was validated as a surrogate for the AA after calibration, venous sampling proved insufficient for IDIF calibration, limiting its utility for precise V_T_ estimation. However, venous samples showed promise for less invasive measurement of plasma-to-whole blood ratios and plasma parent fractions.

## Data Availability

The datasets generated during and/or analyzed during the current study are available from the corresponding author upon reasonable request.
