# Supplementary material for: Evaluating image-derived input functions for cerebral [18F]MC225 PET studies
Source: Front Nucl Med. 2025 Jun 5;5:1597902. doi: 10.3389/fnume.2025.1597902 (PMC12176838; doi:10.3389/fnume.2025.1597902)
Supplement: Supplementary file 1 [file Datasheet1.docx]

**Supplemental Data**

**Results**

Subjects S1 and S3 experienced premature sampling termination at 20 minutes for S1 and 30 minutes for S3. As a result, the tails of the whole blood time-activity curve from the aortic arch (AA) calibrated using the manual arterial samples (BTAC_AA_CAL_) were estimated using the limited time points available, with the last recorded sample value repeated until the end of the scan.

For subjects S1 and S3, the plasma-to-whole blood ratio and plasma parent fraction were estimated based on the available time points.

For the validation of venous and arterial samples using subjects acquired on the Vision PET/CT scanner, one of the six subjects with arterial and venous sampling did not have a plasma parent fraction estimated due to technical difficulties, and another subject lacked plasma parent fraction estimation at 20 minutes. In addition, plasma-to-whole blood ratio estimates were missing for one subject at 5 and 60 minutes, another at 60 minutes, and a third at 5 minutes due to logistical issues.


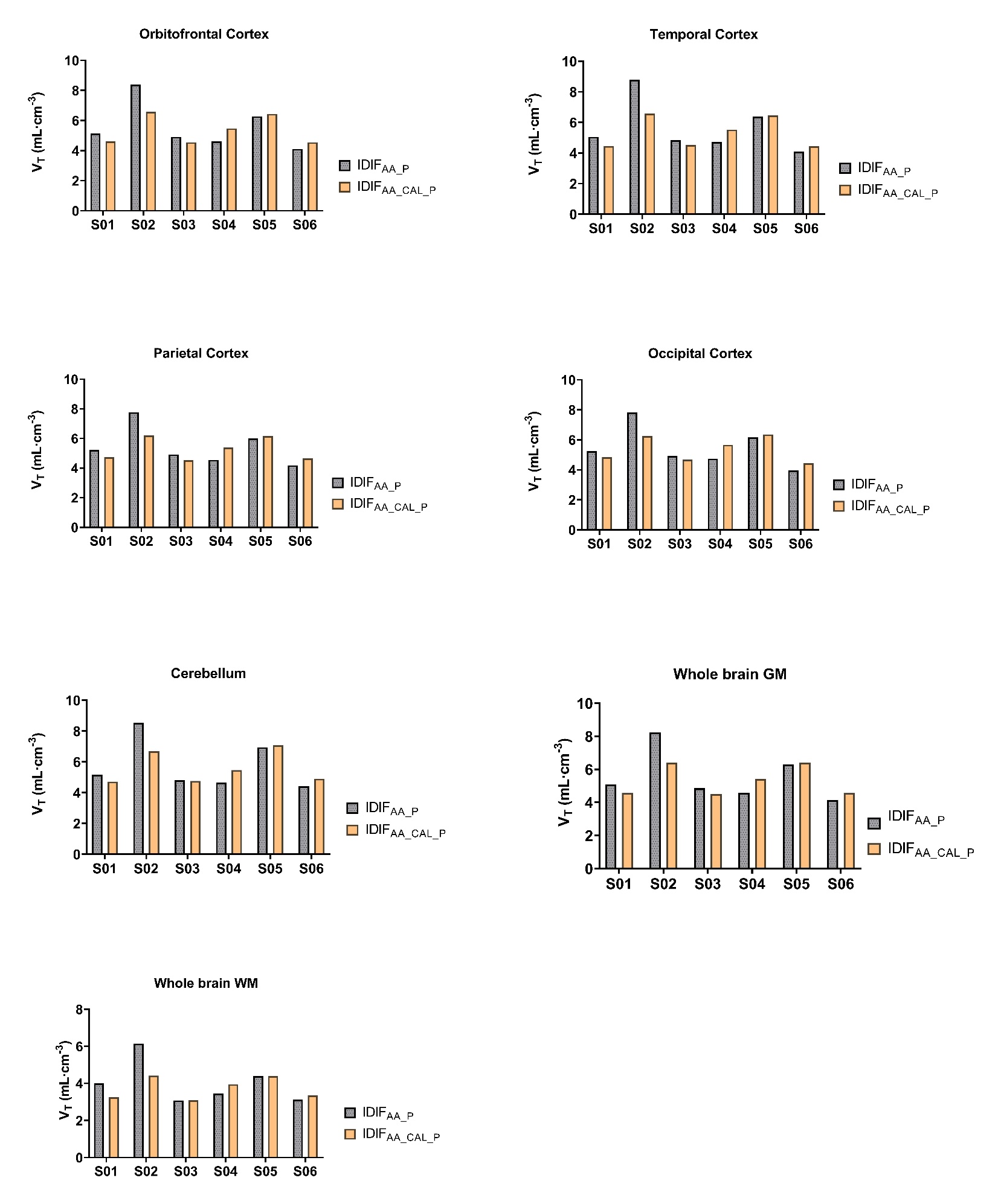


**Supplementary Figure S1.** Individual V_T_ values across brain regions using IDIF_AA_P_ and IDIF_AA_CAL_P._

_
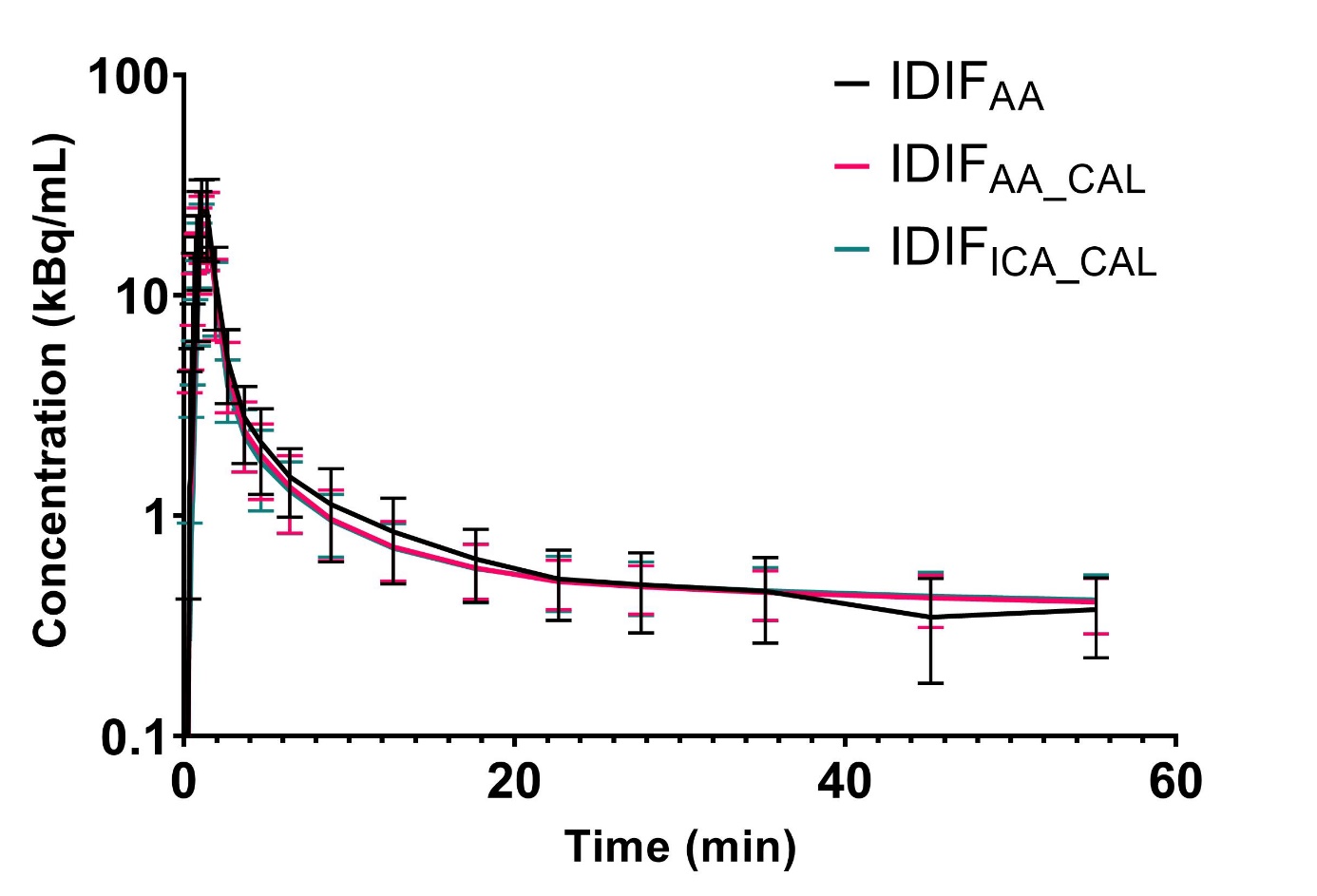
_

**Supplementary Figure S2**: Comparison of whole blood TAC derived from different arterial sources. Plots show IDIF_AA_, IDIF_A_CAL_, and IDIF_ICA_CAL_ across six participants.


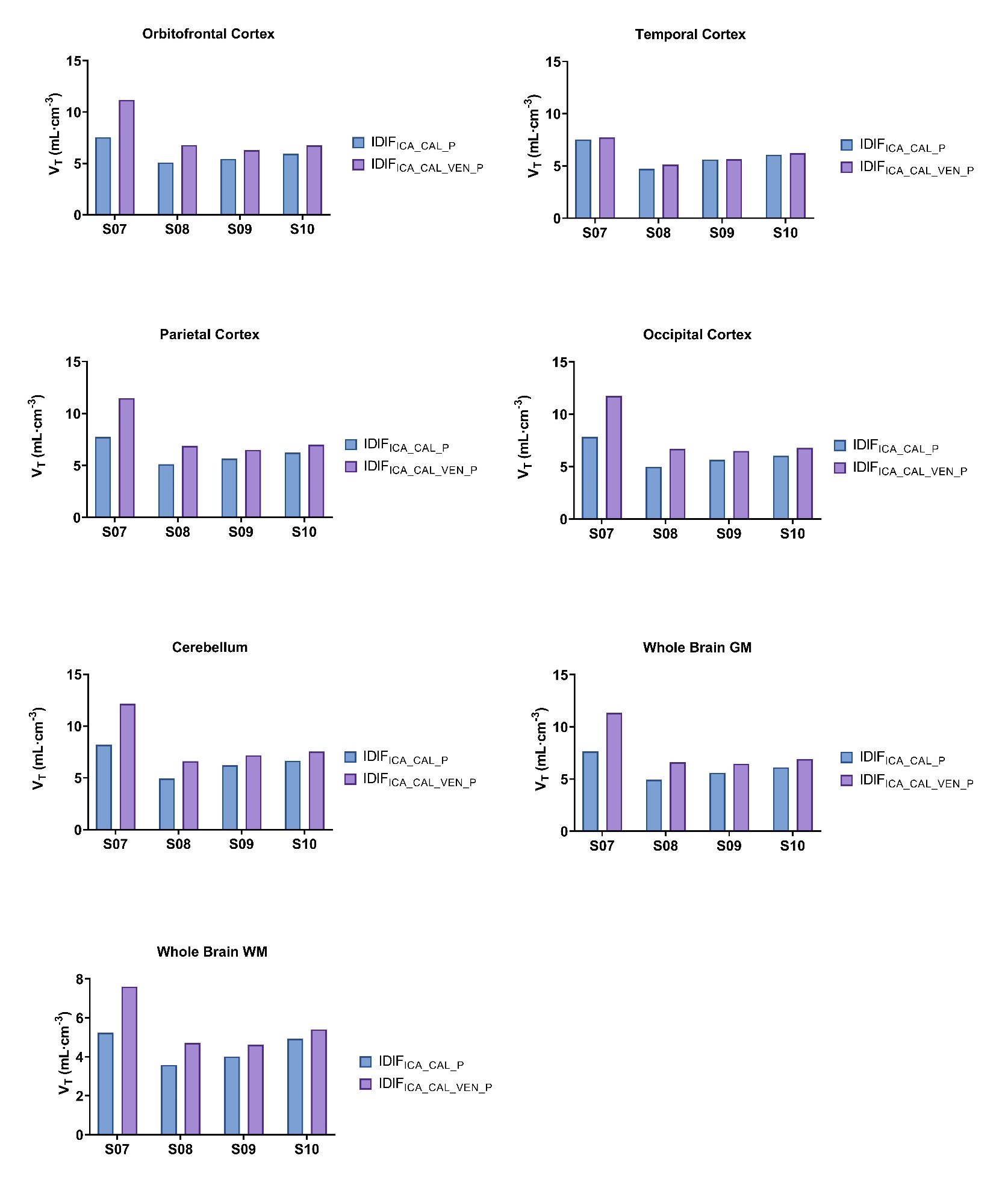


**Supplementary Figure S3.** Individual V_T_ values across brain regions using IDIF_AA_CAL_P_ and IDIF_ICA_CAL_P_.

_
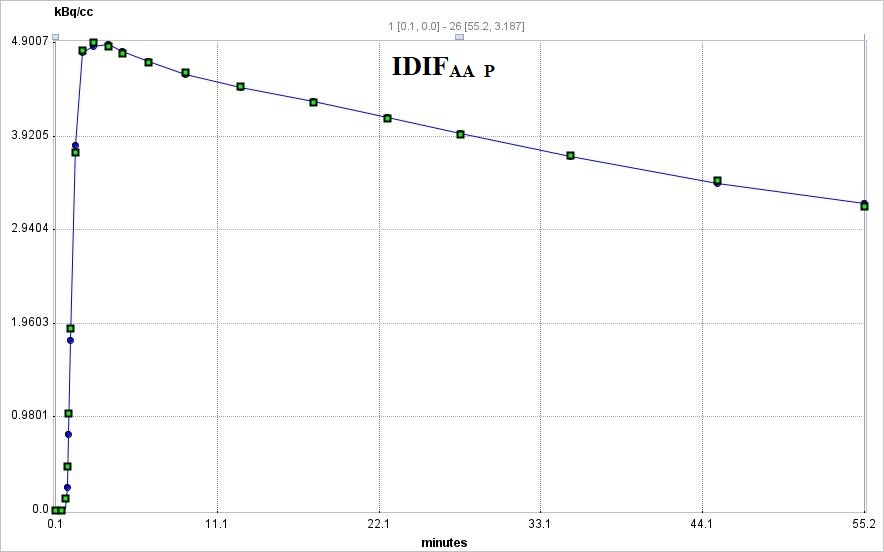
_

(A)

(B)

_
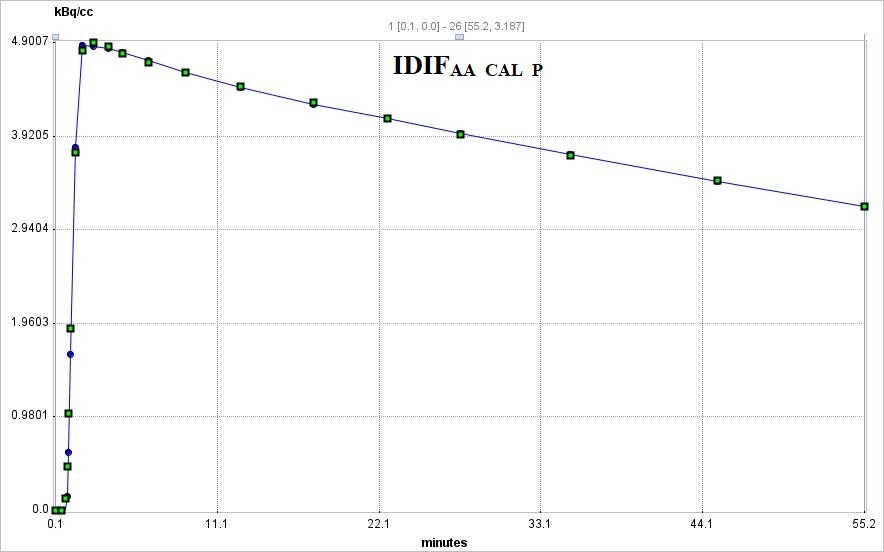
_


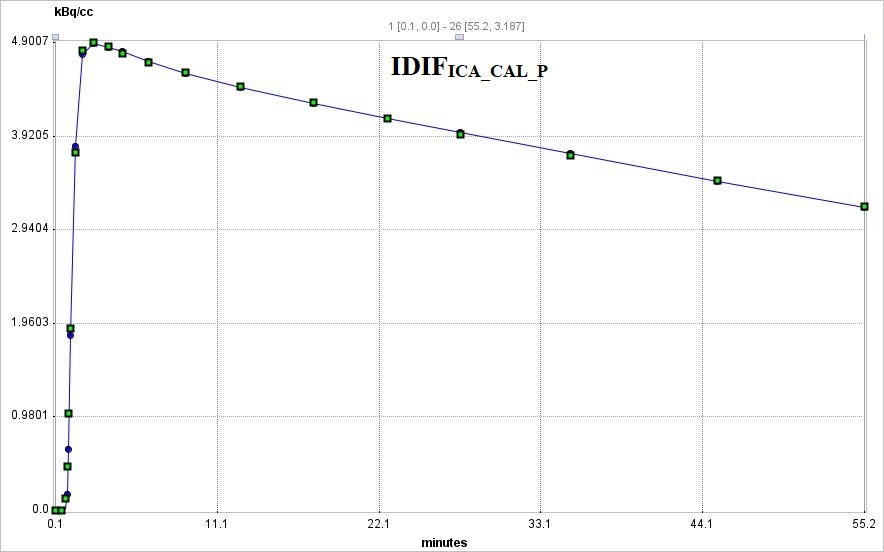


(C)

**Supplementary Figure S4**: Example of whole brain GM tissue time activity curve, from subject SO5, fitted using the IDIFS evaluated in this study: (A) IDIF_AA_P_; (B) IDIF_AA_CAL_P_; (C) IDIF_ICA_CAL_P_.


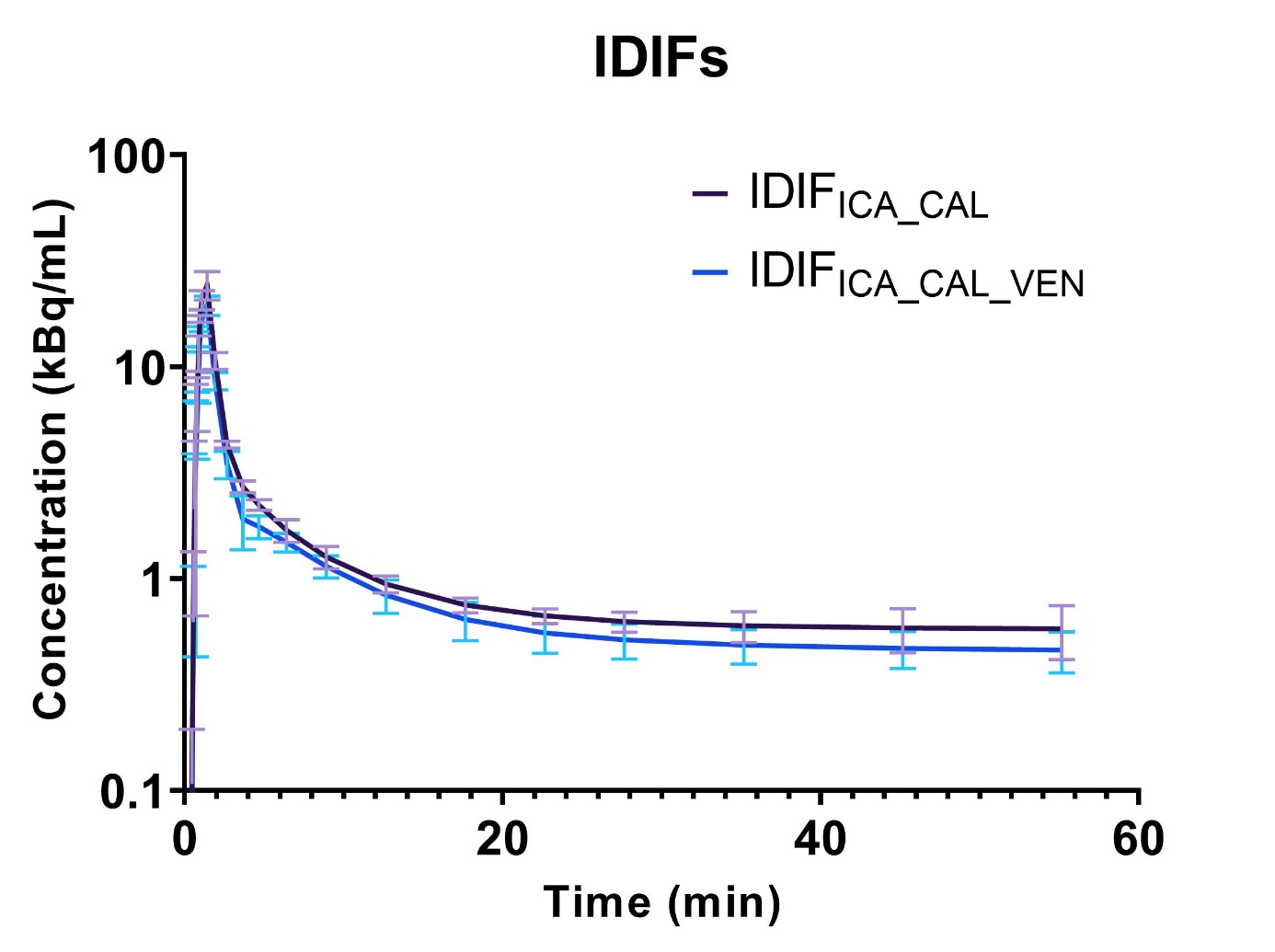


**Supplementary Figure S5.** Comparison of whole blood TAC derived from different arterial sources. Plots show IDIF_ICA_CAL_, and IDIF_CA_CAL_VEN_ across four participants.


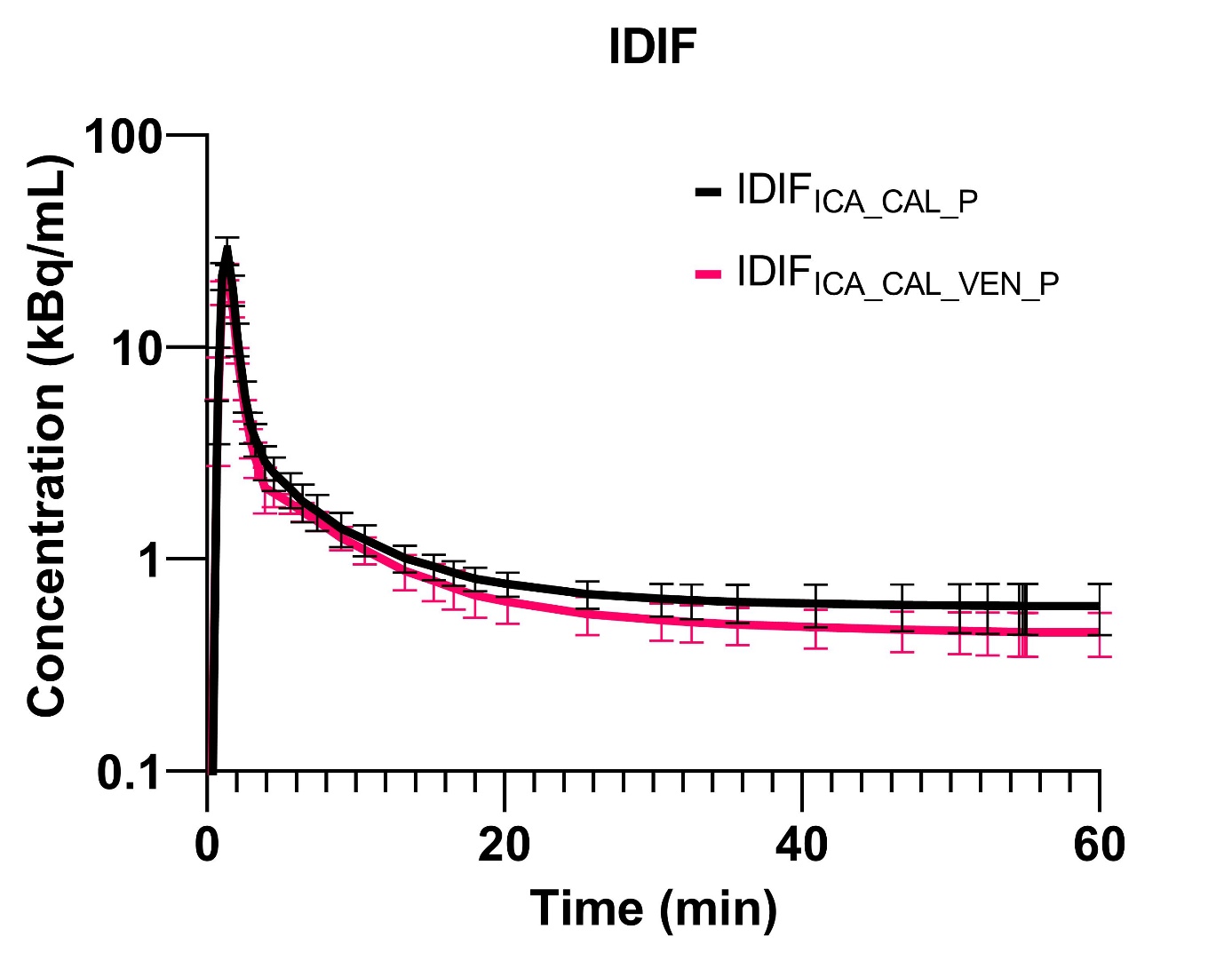


**Supplementary Figure S6.** Comparison of IDIF_ICA_CAL_P_, and IDIF_ICA_CAL_VEN_P_ across four participants.


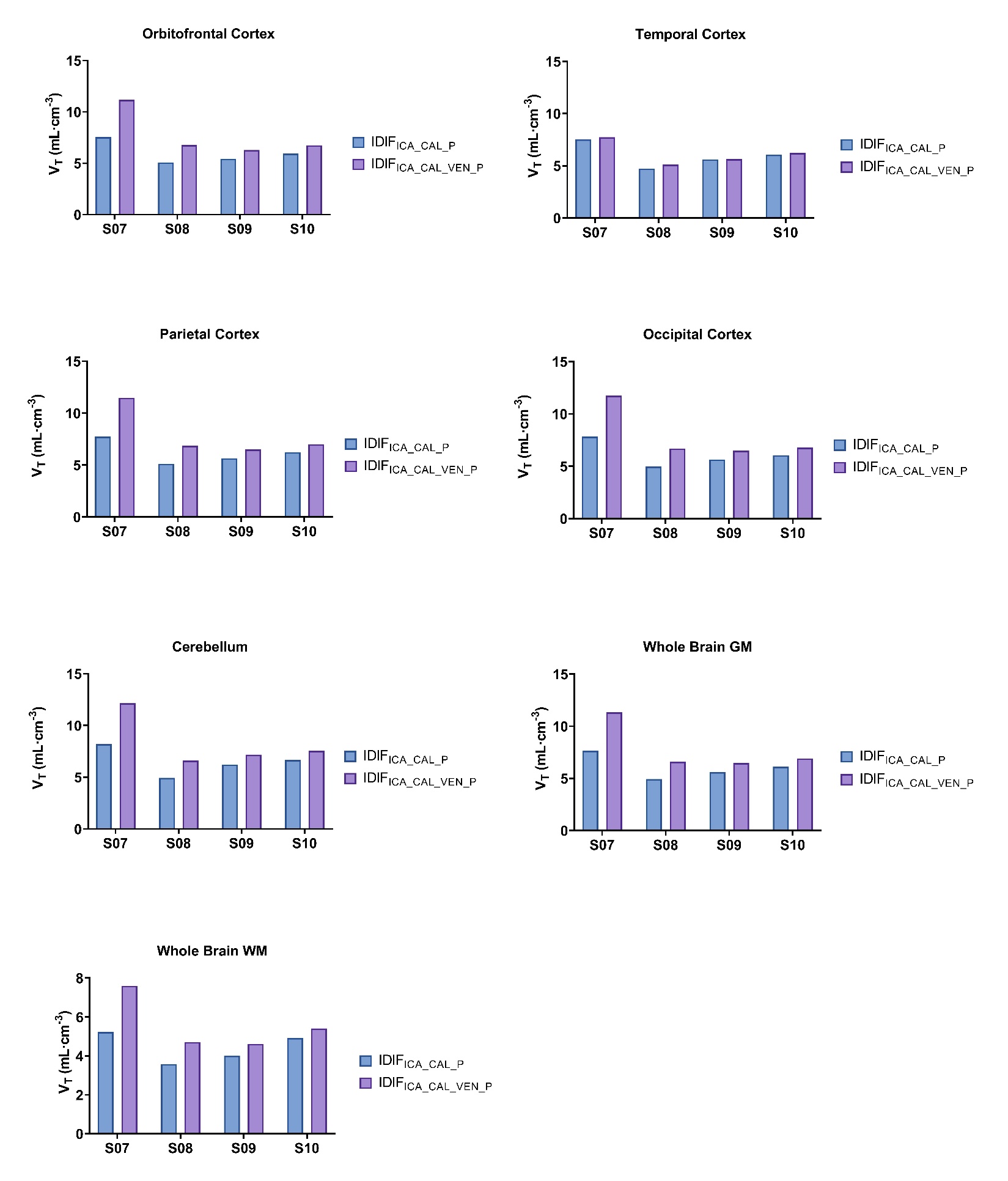


**Supplementary Figure S7**. Individual V_T_ values across brain regions using IDIF_ICA_CAL_P_ and IDIF_ICA_CAL_VEN_P_.

(A)


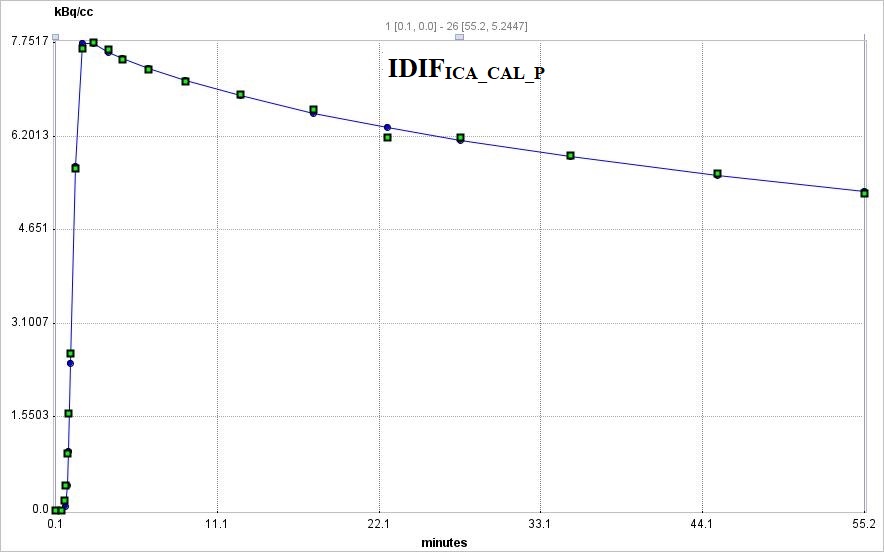


(B)


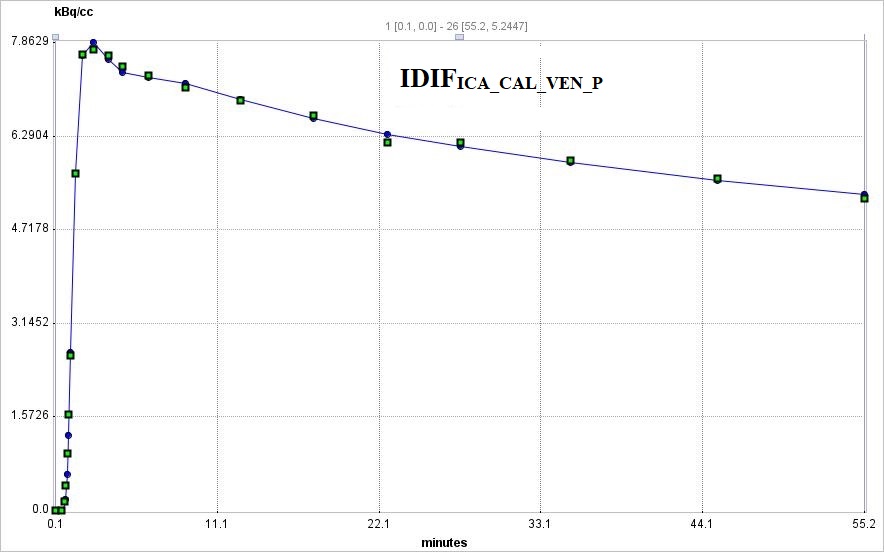


**Supplementary Figure S8**: Whole brain GM tissue time activity curve, from subject SO7, fitted using the IDIFS evaluated in this study: (A) IDIF_ICA_CAL_P_; (B) IDIF_ICA_CAL_VEN_P_.
